# *N*-(Pyrazin-2-yl)-1,8-naphthyridin-2-amine

**DOI:** 10.1107/S160053681300319X

**Published:** 2013-02-06

**Authors:** Yan-Shan Duan, Wen-Zhen Wang, Yuh-Sheng Wen, Yu-Qin Zhu, Shie-Ming Peng

**Affiliations:** a School of Chemistry and Chemical Engineering, Xi’an Shiyou University, Xi’an 710065, People’s Republic of China; b Institute of Chemistry, Academia Sinica, Taipei, Taiwan; c Department of Chemistry, National Taiwan University, Taipei 106, Taiwan

## Abstract

There are two independent mol­ecules in the asymmetric unit of the title compound, C_12_H_9_N_5_, in which the C—N(amine)—C angles differ slightly [129.63 (11) and 132.02 (11)°]. In each independent mol­ecule, an intra­molecular C—H⋯N hydrogen bond stabilizes the mol­ecular structure, forming an *S*(6) ring motif. The independent mol­ecules are linked *via* an N—H⋯N hydrogen bond. Further N—H⋯N and C—H⋯N hydrogen bonds connect the mol­ecules into chains along *c* axis. Pairs of C—H⋯π inter­actions between the chains lead to sheets parallel to the *b* axis. These are linked by π–π inter­actions between the naphthyridine and pyrazine rings [centroid–centroid separations of 3.553 (8) Å] into a three-dimensional supra­molecular network.

## Related literature

For related structures, see: Alvarez-Rua *et al.* (2004 ▶); Basato *et al.* (2006 ▶); Ghosh *et al.* (2010 ▶); Jin *et al.* (2010 ▶, 2011 ▶). For graph-set analysis, see: Bernstein *et al.* (1995 ▶).
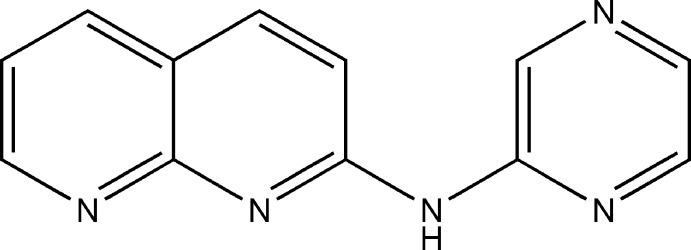


## Experimental

### 

#### Crystal data

C_12_H_9_N_5_
*M* *_r_* = 223.24
Triclinic, 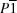

*a* = 7.8608 (3) Å
*b* = 11.8200 (5) Å
*c* = 11.9356 (4) Å
α = 105.096 (2)°
β = 98.086 (2)°
γ = 101.854 (2)°
*V* = 1025.53 (7) Å^3^
*Z* = 4
Mo *K*α radiation
μ = 0.09 mm^−1^
*T* = 100 K
0.28 × 0.2 × 0.18 mm

#### Data collection

Bruker SMART APEX CCD diffractometer
Absorption correction: multi-scan (*SADABS*; Bruker, 2001 ▶) *T*_min_ = 0.927, *T*_max_ = 0.991
16351 measured reflections
3606 independent reflections
2557 reflections with *I* > 2σ(*I*)
*R*_int_ = 0.037

#### Refinement

*R*[*F* ^2^ > 2σ(*F* ^2^)] = 0.031
*wR*(*F* ^2^) = 0.074
*S* = 0.93
3606 reflections
308 parameters
H-atom parameters constrained
Δρ_max_ = 0.18 e Å^−3^
Δρ_min_ = −0.20 e Å^−3^

### 

Data collection: *SMART* (Bruker, 2007 ▶); cell refinement: *SAINT* (Bruker, 2007 ▶); data reduction: *SAINT*; program(s) used to solve structure: *SHELXS97* (Sheldrick, 2008 ▶); program(s) used to refine structure: *SHELXL97* (Sheldrick, 2008 ▶); molecular graphics: *ORTEP-3 for Windows* (Farrugia, 2012 ▶); software used to prepare material for publication: *WinGX* (Farrugia, 2012 ▶).

## Supplementary Material

Crystal structure: contains datablock(s) global, I. DOI: 10.1107/S160053681300319X/rk2390sup1.cif

Structure factors: contains datablock(s) I. DOI: 10.1107/S160053681300319X/rk2390Isup2.hkl

Supplementary material file. DOI: 10.1107/S160053681300319X/rk2390Isup3.cml

Additional supplementary materials:  crystallographic information; 3D view; checkCIF report

## Figures and Tables

**Table 1:** Hydrogen-bond geometry (Å, °) *Cg*1 is the centroid of the N22/C23–C25/C29/C30 ring.

*D*—H⋯*A*	*D*—H	H⋯*A*	*D*⋯*A*	*D*—H⋯*A*
C27—H27⋯N33	0.93	2.33	2.9318 (17)	122
C17—H17⋯N1	0.93	2.24	2.8518 (17)	123
N31—H31⋯N2	0.86	2.14	2.9396 (15)	154
N11—H11⋯N22^i^	0.86	2.23	3.0766 (15)	171
C26—H26⋯N16^ii^	0.93	2.51	3.3608 (17)	152
C15—H15⋯*Cg*1^iii^	0.93	2.74	3.472 (2)	136

Symmetry codes: (i) 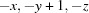; (ii) 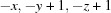; (iii) 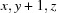.

**Fig. 1.**
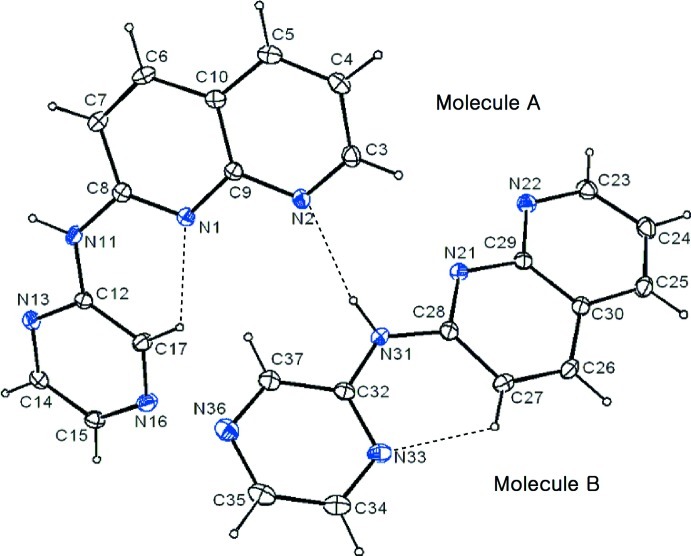
The molecular structure of I with the atom numbering scheme. Displacement ellipsoids are drawn at the 30% probability level. H atoms are presented as a small spheres of arbitrary radius. H bonds are indicated by dashed lines.

**Fig. 2.**
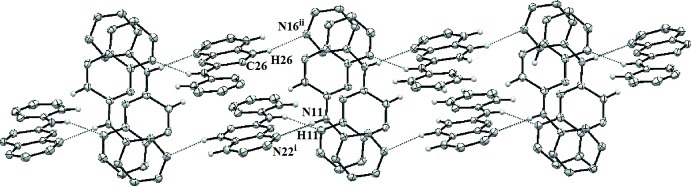
Chains parallel c axis in the crystal structure of I. H bonds are indicated with dashed lines. Symmetry codes: (i) -x, -y+1, -z; (ii) -x, -y+1, -z+1.

**Fig. 3.**
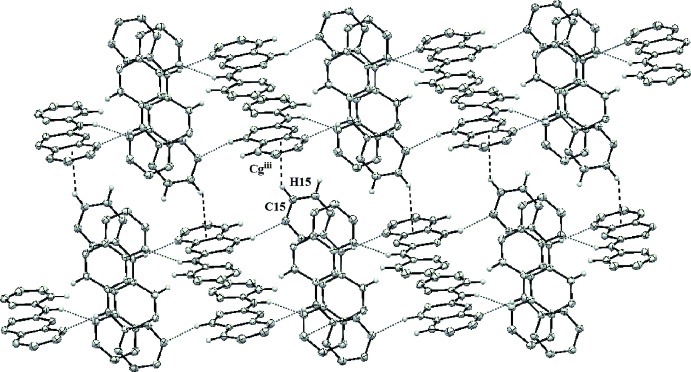
A sheet parallels b axis in the crystal structure of I. H bonds are indicated with dashed lines. Symmetry code: (iii) x, y+1, z.

**Fig. 4.**
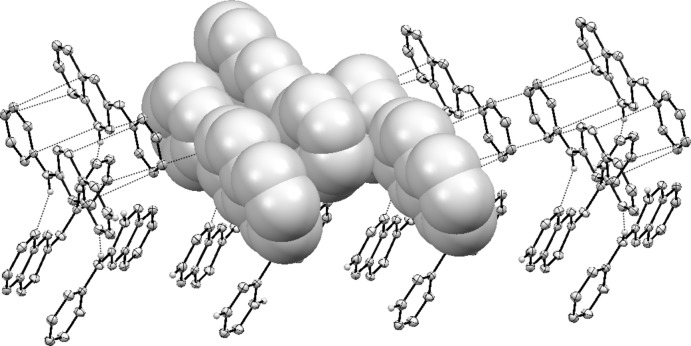
Three dimensional network in the crystal structure of Iviaπ–π interactions between the naphthyridine and pyrazine rings. H bonds are indicated with dashed lines. Naphthyridine and pyrazine rings paired with π–π interaction are presented as spacefilled.

## supplementary crystallographic information

### Comment

1,8-Naphthyridine, a simple heterocycle compound with two ring nitrogen atoms as hydrogen bond acceptors, has been widely used by various groups in the area of molecular recognition research as hydrogen bonding building block. Some interesting coordination polymers assembled with 1,8-naphthyridine have been reported, showing various structural motifs (Alvarez-Rua *et al.*, 2004; Basato *et al.*, 2006; Ghosh *et al.*, 2010; Jin *et al.*, 2010; Jin *et al.*, 2011). The title compound, C_12_H_9_N_5_, I, contains an array of hydrogen bond NH donors and N acceptors and therefore follow different hydrogen bonding packing patterns. In this paper, we report its crystal structure, which crystallizes with two unique molecules, *A* & *B* (Fig. 1), focusing on three-dimensional supramolecular network *via* weak noncovalent interactions.

The molecular structure of the title compound is shown in Fig. 1. The C—N(amine)—C angles of the two chemically equal molecules in the dimer are slightly diferent, showing 129.63 (11)° and 132.02 (11)°, for molecule *A* and *B*, respectively. Two intramolecular hydrogen bonds C27—H27···N33 and C17—H17···N1 (Table 1) stabilize the molecular structure and result in an *S*(6) ring motif (Bernstein *et al.*, 1995). Two independent molecules in the title compound form a molecular pair *via* N31—H31···N2 hydrogen bonds (Fig. 1). N11—H11···N22^i^ intermolecular hydrogen bonds link dimer molecules into rings as basic expanding units, which are joined into one-dimensional chains along *c* axis through C26—H26···N16^ii^ hydrogen bonds (Fig. 2). Pairs of C15—H15···*Cg*^iii^ interactions between the chains construct sheets parallel to *b* axis (Fig. 3). Extensive three dimensional supramolecular networks are formed by π–π interactions between the naphthyridine and pyrazine rings with centroid–centroid separations of 3.553 (8)Å propagating along *a* axis (Fig. 4). Symmetry codes: (i) -*x*, -*y*+1, -*z*; (ii) -*x*, -*y*+1, -*z*+1; (iii) *x*, *y*+1, *z*.

### Experimental

A mixture of 2-chloro-1,8-naphthyridine (8.0 g, 40 mmol), pyrazin-2-amine (4.6 g, 48 mmol), Pd_2_(*dba*)_3_ (0.73 g, 0.80 mmol) (*dba* is dibenzylideneacetone), 1,3-bis(diphenylphosphino)propane (0.66 g, 1.6 mmol) and *Bu^t^*OK (13.1 g, 136 mmol) in dry toluene (350 ml) was refluxed under argon with stirring for 4 days. The crude product was washed with water, benzene and methanol and recrystallized from acetone.

### Refinement

The H atoms attached to C and N atoms were positioned geometrically and refined using in the riding model, with C—H = 0.93 Å, N—H = 0.86 Å and *U*_iso_(H) = 1.2 *U*_eq_(C,N).

### Crystal data

**Table:**

C_12_H_9_N_5_	*Z* = 4
*M**_r_* = 223.24	*F*(000) = 464
Triclinic, *P*1	*D*_x_ = 1.446 Mg m^−^^3^
Hall symbol: -P 1	Mo *K*α radiation, λ = 0.71073 Å
*a* = 7.8608 (3) Å	Cell parameters from 5652 reflections
*b* = 11.8200 (5) Å	θ = 2.7–29.1°
*c* = 11.9356 (4) Å	µ = 0.09 mm^−^^1^
α = 105.096 (2)°	*T* = 100 K
β = 98.086 (2)°	Prism, yellow
γ = 101.854 (2)°	0.28 × 0.2 × 0.18 mm
*V* = 1025.53 (7) Å^3^	

### Data collection

**Table:**

Bruker SMART APEX CCD diffractometer	3606 independent reflections
Radiation source: fine-focus sealed tube	2557 reflections with *I* > 2σ(*I*)
Graphite monochromator	*R*_int_ = 0.037
φ and ω scans	θ_max_ = 25.0°, θ_min_ = 1.8°
Absorption correction: multi-scan (*SADABS*; Bruker, 2001)	*h* = −8→9
*T*_min_ = 0.927, *T*_max_ = 0.991	*k* = −14→14
16351 measured reflections	*l* = −14→14

### Refinement

**Table:**

Refinement on *F*^2^	Secondary atom site location: difference Fourier map
Least-squares matrix: full	Hydrogen site location: inferred from neighbouring sites
*R*[*F*^2^ > 2σ(*F*^2^)] = 0.031	H-atom parameters constrained
*wR*(*F*^2^) = 0.074	*w* = 1/[σ^2^(*F*_o_^2^) + (0.0411*P*)^2^] where *P* = (*F*_o_^2^ + 2*F*_c_^2^)/3
*S* = 0.93	(Δ/σ)_max_ < 0.001
3606 reflections	Δρ_max_ = 0.18 e Å^−^^3^
308 parameters	Δρ_min_ = −0.20 e Å^−^^3^
0 restraints	Extinction correction: *SHELXL97* (Sheldrick, 2008), Fc^*^=kFc[1+0.001xFc^2^λ^3^/sin(2θ)]^-1/4^
Primary atom site location: structure-invariant direct methods	Extinction coefficient: 0.0042 (11)

### Special details

**Table:**

Geometry. All s.u.'s (except the s.u. in the dihedral angle between two l.s. planes) are estimated using the full covariance matrix. The cell s.u.'s are taken into account individually in the estimation of s.u.'s in distances, angles and torsion angles; correlations between s.u.'s in cell parameters are only used when they are defined by crystal symmetry. An approximate (isotropic) treatment of cell s.u.'s is used for estimating s.u.'s involving l.s. planes.
Refinement. Refinement of *F*^2^ against ALL reflections. The weighted *R*-factor *wR* and goodness of fit *S* are based on *F*^2^, conventional *R*-factors *R* are based on *F*, with *F* set to zero for negative *F*^2^. The threshold expression of *F*^2^ > σ(*F*^2^) is used only for calculating *R*-factors(gt) *etc*. and is not relevant to the choice of reflections for refinement. *R*-factors based on *F*^2^ are statistically about twice as large as those based on *F*, and *R*-factors based on ALL data will be even larger.

### Fractional atomic coordinates and isotropic or equivalent isotropic displacement parameters (Å^2^)

**Table:**

	*x*	*y*	*z*	*U*_iso_*/*U*_eq_	
N1	0.22552 (13)	0.57902 (10)	0.04843 (9)	0.0189 (3)	
N2	0.21678 (14)	0.40952 (10)	0.10986 (9)	0.0201 (3)	
N11	0.22899 (14)	0.75053 (10)	−0.01282 (9)	0.0209 (3)	
H11	0.2565	0.7845	−0.0656	0.025*	
N13	0.14424 (14)	0.92338 (10)	0.06217 (10)	0.0222 (3)	
N16	0.09496 (15)	0.86566 (11)	0.26882 (10)	0.0260 (3)	
N21	−0.04515 (14)	0.29804 (9)	0.29392 (9)	0.0196 (3)	
N22	−0.27965 (14)	0.13223 (10)	0.21168 (10)	0.0234 (3)	
N31	0.18130 (14)	0.46608 (10)	0.35900 (9)	0.0213 (3)	
H31	0.1625	0.4602	0.2847	0.026*	
N33	0.36229 (14)	0.58552 (10)	0.54390 (10)	0.0238 (3)	
N36	0.52942 (15)	0.73882 (10)	0.42486 (10)	0.0277 (3)	
C3	0.23662 (17)	0.29818 (12)	0.09116 (12)	0.0230 (3)	
H3	0.2164	0.2607	0.1493	0.028*	
C4	0.28551 (17)	0.23258 (12)	−0.00894 (12)	0.0238 (3)	
H4	0.2998	0.1550	−0.0162	0.029*	
C5	0.31171 (17)	0.28572 (12)	−0.09594 (12)	0.0232 (3)	
H5	0.3434	0.2444	−0.1642	0.028*	
C6	0.31156 (17)	0.46677 (13)	−0.16631 (12)	0.0229 (3)	
H6	0.3405	0.4303	−0.2376	0.027*	
C7	0.28987 (17)	0.57976 (13)	−0.14393 (12)	0.0227 (3)	
H7	0.3026	0.6219	−0.1993	0.027*	
C8	0.24673 (16)	0.63419 (12)	−0.03287 (12)	0.0188 (3)	
C9	0.24464 (16)	0.46328 (12)	0.02396 (11)	0.0177 (3)	
C10	0.29047 (16)	0.40294 (12)	−0.08155 (11)	0.0190 (3)	
C12	0.17354 (17)	0.82116 (12)	0.07953 (11)	0.0182 (3)	
C14	0.08721 (18)	0.99392 (12)	0.14756 (12)	0.0237 (3)	
H14	0.0626	1.0647	0.1374	0.028*	
C15	0.06335 (18)	0.96630 (12)	0.24991 (12)	0.0235 (3)	
H15	0.0243	1.0189	0.3072	0.028*	
C17	0.14880 (18)	0.79278 (13)	0.18385 (11)	0.0234 (3)	
H17	0.1705	0.7213	0.1939	0.028*	
C23	−0.38828 (18)	0.03571 (13)	0.22223 (13)	0.0261 (4)	
H23	−0.4718	−0.0141	0.1553	0.031*	
C24	−0.38621 (19)	0.00346 (13)	0.32681 (13)	0.0281 (4)	
H24	−0.4634	−0.0670	0.3282	0.034*	
C25	−0.26920 (18)	0.07709 (12)	0.42639 (13)	0.0257 (4)	
H25	−0.2672	0.0584	0.4975	0.031*	
C26	−0.02570 (17)	0.26653 (12)	0.51840 (12)	0.0224 (3)	
H26	−0.0175	0.2559	0.5933	0.027*	
C27	0.08325 (18)	0.36368 (12)	0.50309 (12)	0.0223 (3)	
H27	0.1639	0.4214	0.5670	0.027*	
C28	0.07114 (17)	0.37501 (12)	0.38708 (12)	0.0191 (3)	
C29	−0.15825 (17)	0.20387 (12)	0.31086 (12)	0.0194 (3)	
C30	−0.15148 (18)	0.18136 (12)	0.42162 (12)	0.0201 (3)	
C32	0.31538 (17)	0.56440 (12)	0.42761 (12)	0.0188 (3)	
C34	0.49447 (18)	0.68540 (13)	0.60011 (13)	0.0268 (4)	
H34	0.5317	0.7046	0.6818	0.032*	
C35	0.57667 (19)	0.76004 (13)	0.54274 (13)	0.0291 (4)	
H35	0.6679	0.8275	0.5865	0.035*	
C37	0.39957 (17)	0.64185 (12)	0.36948 (13)	0.0228 (3)	
H37	0.3617	0.6237	0.2880	0.027*	

### Atomic displacement parameters (Å^2^)

**Table:**

	*U*^11^	*U*^22^	*U*^33^	*U*^12^	*U*^13^	*U*^23^
N1	0.0177 (6)	0.0199 (7)	0.0191 (7)	0.0044 (5)	0.0036 (5)	0.0062 (6)
N2	0.0206 (7)	0.0181 (7)	0.0208 (7)	0.0028 (5)	0.0040 (5)	0.0064 (5)
N11	0.0256 (7)	0.0230 (7)	0.0192 (6)	0.0083 (5)	0.0100 (5)	0.0103 (6)
N13	0.0249 (7)	0.0188 (7)	0.0238 (7)	0.0058 (5)	0.0058 (6)	0.0072 (6)
N16	0.0345 (7)	0.0259 (7)	0.0207 (7)	0.0129 (6)	0.0076 (6)	0.0071 (6)
N21	0.0220 (7)	0.0188 (7)	0.0187 (6)	0.0062 (5)	0.0059 (5)	0.0051 (6)
N22	0.0243 (7)	0.0218 (7)	0.0230 (7)	0.0043 (6)	0.0071 (6)	0.0049 (6)
N31	0.0258 (7)	0.0228 (7)	0.0155 (6)	0.0050 (6)	0.0051 (5)	0.0067 (5)
N33	0.0217 (7)	0.0272 (7)	0.0213 (7)	0.0096 (6)	0.0043 (6)	0.0027 (6)
N36	0.0244 (7)	0.0252 (7)	0.0313 (8)	0.0060 (6)	0.0050 (6)	0.0049 (6)
C3	0.0210 (8)	0.0208 (9)	0.0249 (8)	0.0006 (7)	0.0022 (7)	0.0083 (7)
C4	0.0205 (8)	0.0186 (8)	0.0285 (9)	0.0038 (6)	0.0014 (7)	0.0034 (7)
C5	0.0179 (8)	0.0242 (9)	0.0227 (8)	0.0056 (7)	0.0026 (6)	−0.0006 (7)
C6	0.0208 (8)	0.0321 (9)	0.0157 (8)	0.0093 (7)	0.0056 (6)	0.0042 (7)
C7	0.0230 (8)	0.0291 (9)	0.0194 (8)	0.0085 (7)	0.0069 (7)	0.0098 (7)
C8	0.0136 (7)	0.0221 (8)	0.0201 (8)	0.0041 (6)	0.0016 (6)	0.0066 (7)
C9	0.0129 (7)	0.0193 (8)	0.0180 (8)	0.0011 (6)	0.0002 (6)	0.0046 (6)
C10	0.0134 (7)	0.0216 (8)	0.0186 (8)	0.0030 (6)	0.0007 (6)	0.0028 (7)
C12	0.0157 (7)	0.0184 (8)	0.0183 (8)	0.0031 (6)	0.0017 (6)	0.0040 (6)
C14	0.0252 (8)	0.0175 (8)	0.0269 (9)	0.0053 (7)	0.0039 (7)	0.0051 (7)
C15	0.0264 (8)	0.0209 (8)	0.0220 (8)	0.0082 (7)	0.0043 (7)	0.0031 (7)
C17	0.0302 (9)	0.0248 (9)	0.0188 (8)	0.0127 (7)	0.0063 (7)	0.0075 (7)
C23	0.0252 (8)	0.0226 (8)	0.0293 (9)	0.0052 (7)	0.0097 (7)	0.0041 (7)
C24	0.0302 (9)	0.0226 (9)	0.0349 (9)	0.0062 (7)	0.0147 (8)	0.0106 (8)
C25	0.0324 (9)	0.0271 (9)	0.0259 (9)	0.0132 (7)	0.0140 (7)	0.0132 (7)
C26	0.0276 (8)	0.0285 (9)	0.0184 (8)	0.0141 (7)	0.0097 (7)	0.0112 (7)
C27	0.0242 (8)	0.0269 (9)	0.0178 (8)	0.0108 (7)	0.0050 (6)	0.0064 (7)
C28	0.0208 (8)	0.0189 (8)	0.0218 (8)	0.0103 (7)	0.0080 (7)	0.0071 (7)
C29	0.0212 (8)	0.0182 (8)	0.0221 (8)	0.0092 (6)	0.0091 (7)	0.0059 (7)
C30	0.0228 (8)	0.0210 (8)	0.0219 (8)	0.0113 (7)	0.0100 (7)	0.0083 (7)
C32	0.0174 (8)	0.0183 (8)	0.0205 (8)	0.0086 (6)	0.0038 (6)	0.0023 (7)
C34	0.0210 (8)	0.0309 (9)	0.0245 (9)	0.0096 (7)	0.0024 (7)	0.0003 (7)
C35	0.0217 (8)	0.0259 (9)	0.0326 (10)	0.0058 (7)	0.0023 (7)	−0.0012 (7)
C37	0.0217 (8)	0.0228 (8)	0.0241 (8)	0.0081 (7)	0.0043 (7)	0.0059 (7)

### Geometric parameters (Å, º)

**Table:**

N1—C8	1.3134 (15)	C6—C7	1.3434 (18)
N1—C9	1.3669 (16)	C6—C10	1.4190 (17)
N2—C3	1.3218 (16)	C6—H6	0.9300
N2—C9	1.3596 (15)	C7—C8	1.4346 (18)
N11—C8	1.3727 (16)	C7—H7	0.9300
N11—C12	1.3810 (16)	C9—C10	1.4105 (18)
N11—H11	0.8600	C12—C17	1.4000 (17)
N13—C14	1.3321 (17)	C14—C15	1.3715 (17)
N13—C12	1.3368 (16)	C14—H14	0.9300
N16—C17	1.3286 (16)	C15—H15	0.9300
N16—C15	1.3316 (16)	C17—H17	0.9300
N21—C28	1.3211 (16)	C23—C24	1.3957 (19)
N21—C29	1.3534 (15)	C23—H23	0.9300
N22—C23	1.3234 (16)	C24—C25	1.3568 (19)
N22—C29	1.3628 (16)	C24—H24	0.9300
N31—C32	1.3748 (16)	C25—C30	1.4005 (18)
N31—C28	1.3812 (15)	C25—H25	0.9300
N31—H31	0.8600	C26—C27	1.3545 (18)
N33—C32	1.3277 (16)	C26—C30	1.4105 (18)
N33—C34	1.3456 (17)	C26—H26	0.9300
N36—C37	1.3143 (16)	C27—C28	1.4174 (17)
N36—C35	1.3459 (17)	C27—H27	0.9300
C3—C4	1.3947 (19)	C29—C30	1.4108 (17)
C3—H3	0.9300	C32—C37	1.4030 (17)
C4—C5	1.3645 (18)	C34—C35	1.3674 (19)
C4—H4	0.9300	C34—H34	0.9300
C5—C10	1.3987 (19)	C35—H35	0.9300
C5—H5	0.9300	C37—H37	0.9300
			
C8—N1—C9	117.38 (11)	C15—C14—H14	118.7
C3—N2—C9	117.13 (11)	N16—C15—C14	121.31 (13)
C8—N11—C12	129.63 (11)	N16—C15—H15	119.3
C8—N11—H11	115.2	C14—C15—H15	119.3
C12—N11—H11	115.2	N16—C17—C12	121.52 (13)
C14—N13—C12	116.49 (12)	N16—C17—H17	119.2
C17—N16—C15	117.14 (12)	C12—C17—H17	119.2
C28—N21—C29	117.94 (11)	N22—C23—C24	124.57 (14)
C23—N22—C29	116.70 (12)	N22—C23—H23	117.7
C32—N31—C28	132.02 (11)	C24—C23—H23	117.7
C32—N31—H31	114.0	C25—C24—C23	118.61 (13)
C28—N31—H31	114.0	C25—C24—H24	120.7
C32—N33—C34	115.12 (12)	C23—C24—H24	120.7
C37—N36—C35	115.33 (12)	C24—C25—C30	119.71 (13)
N2—C3—C4	125.08 (13)	C24—C25—H25	120.1
N2—C3—H3	117.5	C30—C25—H25	120.1
C4—C3—H3	117.5	C27—C26—C30	120.52 (12)
C5—C4—C3	117.96 (13)	C27—C26—H26	119.7
C5—C4—H4	121.0	C30—C26—H26	119.7
C3—C4—H4	121.0	C26—C27—C28	118.26 (13)
C4—C5—C10	119.43 (13)	C26—C27—H27	120.9
C4—C5—H5	120.3	C28—C27—H27	120.9
C10—C5—H5	120.3	N21—C28—N31	112.89 (11)
C7—C6—C10	120.20 (12)	N21—C28—C27	123.37 (12)
C7—C6—H6	119.9	N31—C28—C27	123.73 (13)
C10—C6—H6	119.9	N21—C29—N22	114.31 (11)
C6—C7—C8	118.70 (13)	N21—C29—C30	123.03 (12)
C6—C7—H7	120.7	N22—C29—C30	122.66 (12)
C8—C7—H7	120.7	C25—C30—C26	125.60 (12)
N1—C8—N11	119.77 (12)	C25—C30—C29	117.65 (13)
N1—C8—C7	123.49 (13)	C26—C30—C29	116.75 (12)
N11—C8—C7	116.74 (12)	N33—C32—N31	121.43 (12)
N2—C9—N1	114.86 (11)	N33—C32—C37	121.48 (13)
N2—C9—C10	121.80 (12)	N31—C32—C37	117.09 (12)
N1—C9—C10	123.33 (12)	N33—C34—C35	123.07 (13)
C5—C10—C9	118.57 (12)	N33—C34—H34	118.5
C5—C10—C6	124.57 (12)	C35—C34—H34	118.5
C9—C10—C6	116.86 (12)	N36—C35—C34	121.93 (14)
N13—C12—N11	113.96 (11)	N36—C35—H35	119.0
N13—C12—C17	120.91 (12)	C34—C35—H35	119.0
N11—C12—C17	125.13 (12)	N36—C37—C32	123.07 (13)
N13—C14—C15	122.60 (13)	N36—C37—H37	118.5
N13—C14—H14	118.7	C32—C37—H37	118.5

### Hydrogen-bond geometry (Å, º)

*Cg*1 is the centroid of the N22/C23–C25/C29/C30 ring.

**Table:**

*D*—H···*A*	*D*—H	H···*A*	*D*···*A*	*D*—H···*A*
C27—H27···N33	0.93	2.33	2.9318 (17)	122
C17—H17···N1	0.93	2.24	2.8518 (17)	123
N31—H31···N2	0.86	2.14	2.9396 (15)	154
N11—H11···N22^i^	0.86	2.23	3.0766 (15)	171
C26—H26···N16^ii^	0.93	2.51	3.3608 (17)	152
C15—H15···*Cg*1^iii^	0.93	2.74	3.472 (2)	136

Symmetry codes: (i) −*x*, −*y*+1, −*z*; (ii) −*x*, −*y*+1, −*z*+1; (iii) *x*, *y*+1, *z*.
